# Boosting Copper Biocidal Activity by Silver Decoration and Few‐Layer Graphene in Coatings on Textile Fibers

**DOI:** 10.1002/gch2.202300113

**Published:** 2023-09-13

**Authors:** Danaja Štular, Nigel Van de Velde, Ana Drinčić, Polona Kogovšek, Arijana Filipić, Katja Fric, Barbara Simončič, Brigita Tomšič, Raghuraj S. Chouhan, Sivasambu Bohm, Suresh Kr. Verma, Pritam Kumar Panda, Ivan Jerman

**Affiliations:** ^1^ National Institute of Chemistry Hajdrihova 19 Ljubljana 1001 Slovenia; ^2^ National Institute of Biology Večna pot 111 Ljubljana 1000 Slovenia; ^3^ Faculty of Natural Sciences and Engineering University of Ljubljana Aškerčeva 12 Ljubljana 1000 Slovenia; ^4^ Institute “Jožef Stefan” Department of Environmental Sciences Jamova 39 Ljubljana 1000 Slovenia; ^5^ Imperial College London South Kensington Campus London SW7 2AZ UK; ^6^ Ångströmlaboratoriet Lägerhyddsv 1 Box 530 Uppsala 75121 Sweden; ^7^ School of Biotechnology KIIT University Bhubaneswar 751024 India

**Keywords:** antibacterial, antiviral, copper micro flakes, few‐layer graphene, pathogen‐repelling coating

## Abstract

The outbreak of the Coronavirus disease 2019 (COVID‐19) pandemic has highlighted the importance of developing antiviral surface coatings that are capable of repelling pathogens and neutralizing them through self‐sanitizing properties. In this study, a novel coating design based on few‐layer graphene (FLG) is proposed and silver‐decorated micro copper flakes (CuMF) that exhibit both antibacterial and antiviral properties. The role of sacrificial anode surfaces and intrinsic graphene defects in enhancing the release of metal ions from CuMF embedded in water‐based binders is investigated. In silico analysis is conducted to better understand the molecular interactions of pathogen‐repelling species with bacterial or bacteriophage proteins. The results show that the optimal amount of CuMF/FLG in the coating leads to a significant reduction in bacterial growth, with reductions of 3.17 and 9.81 log for *Staphylococcus aureus* and *Escherichia coli*, respectively. The same coating also showed high antiviral efficacy, reducing *bacteriophage phi6* by 5.53 log. The antiviral efficiency of the coating is find to be doubled compared to either micro copper flakes or few‐layer graphene alone. This novel coating design is versatile and can be applied to various substrates, such as personal protective clothing and face masks, to provide biocidal activity against both bacterial and viral pathogens.

## Graphical abstract

Schematic presentation of Cu ions formation on textile.

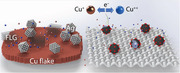

## 1 Introduction

The ongoing pandemic has raised great concern about viruses and other pathogens. The causative agents of respiratory diseases, such as Coronavirus disease 2019 (COVID‐19), are transmitted from an infected person to other hosts in the form of droplets of respiratory fluid. These droplets can be transmitted by sneezing, coughing, or even talking. The pathogens can infect the new host by inhalation or deposition on mucous membranes, especially if the infected person and the new host are in close physical contact. In addition, pathogens can also reach the new host indirectly through droplets deposited on various solid surfaces, where they can be ingested and passed to the host through the nose, mouth, or eyes.^[^ ^1^, ^2^, ^3^ ^]^ This is especially true for textile materials, as textile fibers provide a large surface area for pathogen adherence and growth and therefore pose a high risk for the spread of viral infections. Therefore, the development of personal protective equipment, including protective clothing, that can prevent the transmission of pathogens before they reach a new host is urgently needed.

Surface physical properties, i.e., porosity, adsorption through van der Waals and electrostatic interactions, and surface hydrophobicity play an important role in bacterial and viral persistence.^[^ ^4^, ^5^ ^]^ The wetting properties of the outer layers of proteins in virus capsids can affect their interactions with solid surfaces and the environment. It was demonstrated that the sorption of hydrophobic viruses, i.e., viruses carrying hydrophobic protein outer layers, was favored by surfaces coated with hydrophobic sorbents, while the sorption of hydrophilic viruses was favored by hydrophilic surfaces.^[^ ^6^ ^]^ Most bacteria, e.g., *Staphylococci*, express a broad range of surface proteins involved in their adhesion to an extracellular matrix (ECM), plasma proteins, or directly to host cells.^[^ ^7^ ^]^ However, despite the nature of the fiber surface, pathogens could form a biofilm on the coatings over time,^[^ ^8^ ^]^ which could serve as a breeding ground for other pathogens and in this way negate the protective properties of such a functionalized textile material.

There are two strategies, i.e., passive and active, to provide textiles with antipathogenic properties. The first means of providing protection can be achieved by applying a low adhesion coating on the fiber surface, which could significantly reduce the adsorption ability of pathogens, thus providing passive antimicrobial protection by physically blocking respiratory droplets. However, as pathogen particles are large and have a large variety of surface proteins, there are still multiple patches of positive and negative charges in the *pH* range where pathogens have a stable combination of passive and active antimicrobial properties that should be used for successful protection.^[^ ^9^ ^]^ The second strategy demands the incorporation of metal ions or organic molecules that are harmful to bacteria or viruses.

Copper (Cu) and silver (Ag), in addition to other heavy and coinage metals,^[^ ^10^ ^]^ have been known as active antimicrobial agents for centuries; however, in the medical field, these metals have experienced a renaissance over the last few years due to the increasing emergence of antibiotic‐resistant microorganisms. Cu is a readily available, inexpensive metal that was recognized by the Environmental Protection Agency (EPA) as the first effective metallic antimicrobial agent and is also considered elsewhere as an efficient means of providing textile substrates with antibacterial and antiviral protection.^[^ ^3^, ^11^, ^12^, ^13^ ^]^ Thus, the biocidal effect of Cu surfaces is well known and is based on “contact killing”, which works by dissolving and releasing Cu^+^ and Cu^2+^ ions from the surface, causing oxidative stress at the cell membrane through the production of reactive oxygen species (ROS).^[^ ^14^ ^]^ ROS can generally attack all types of organic materials, including lipids, proteins, and nucleic acids,^[^ ^15^ ^]^ which are the building blocks of all (micro)organisms. However, the high reactivity of the metal particles may promote their agglomeration if used in nanoforms and thus reduce their biocidal activity. This was solved through the addition of support materials such as graphene, which can be used to stabilize the metal particles and thus prevent their agglomeration.^[^ ^16^, ^17^ ^]^ Furthermore, the combined use of Cu and carbon allotropes has already shown promising results in terms of antibacterial activity, and excellent reduction of bacteria was achieved when graphene oxide (GO) was used in combination with CuO^[^ ^18^ ^]^ or Cu^[^ ^19^ ^]^ nanoparticles. Perdikaki et al.^[^ ^20^ ^]^ used graphene as a support for Ag/Cu nanoparticles, and the enhanced antibacterial activity of Ag/Cu nanoparticles was observed due to the synergistic antibacterial effect. Meanwhile, reduced graphene oxide (rGO) was used in combination with Cu nanoparticles and polydopamine on a polyacrylonitrile ultrafiltration sheet membrane to develop membranes with strong antibacterial properties and targeted separation abilities.^[^ ^21^ ^]^ In addition, the antiviral activity of Cu‐graphene nanocomposites was investigated by Das Jana et al.,^[^ ^16^ ^]^ and efficient inactivation of influenza A virus was achieved 30 min after contact with the coating of a polyvinyl alcohol (PVA) matrix incorporated by Cu_2_O nanoparticles/graphene nanocomposites. Moreover, graphene is also known for its antibacterial and antiviral protective properties^[^ ^22^, ^23^, ^24^ ^]^ and could contribute to increasing the antibacterial activity of Cu by enhancing the oxidative stress on microbes.^[^ ^25^ ^]^ Furthermore, previous studies indicate that the absence of the antibacterial effects of pure Ag and Cu nanopatches are due to insufficient ion release from these structures, while the combination of Ag and Pt as well as Ag and Cu leads to enhanced antibacterial activity based on the electrochemically driven enhanced dissolution of Ag and Cu, respectively.^[^ ^26^ ^]^

The pursuit of textiles with exceptional antibacterial, antiviral, and self‐sterilizing properties has been a long‐standing goal in the industry. With the recent advancement of easy‐to‐apply coatings made from a combination of copper, silver, and graphene derivatives, this goal is now within reach. Our research findings reveal that the use of costly physical vapor or chemical deposition processes is not required for outstanding protection, as the multifunctional coating can be prepared from Food and Drug Administration (FDA)‐approved commercial products, making it a cost‐effective solution for enhancing protection against pathogens. Despite this progress, the interaction and antipathogen function of these coatings is largely unknown, calling for a scientific approach to fully understand their effectiveness. In this study, we delve into the potential of a unique coating consisting of micro silver‐decorated copper flakes with the addition of few layers of graphene (FLG) embedded in a water‐based binder. This combination creates an enriched source of Cu^+^ ions, based on two concepts enhancing the efficacy of the coating. The first one is enhanced by release kinetics based on an electrochemical sacrificial anode mechanism where the contact of two materials (Cu/Ag) with different electro potentials is present. The addition of Ag (more Noble material) on the copper surface enables the concept of Cu sacrificial anode and enhanced release of Cu ion species as opposed to using pure Cu species. The second concept revolves around the interaction between the copper and the edge of the FLG. A hydrophobic matrix made from a water‐borne siloxane is then applied to optimize passive protection, providing a pleasant feel when in contact with the skin. Through a comprehensive evaluation using advanced analytical techniques such as scanning electron microscopy (SEM), Raman spectroscopy, Atomic Force Microscopy (AFM), and infrared spectroscopy, we determine the antimicrobial and antiviral efficacy of the coatings, and analyze the wetting properties and biofilm formation potential in conjunction within silico computer analysis. Our results set the foundation for the creation of highly effective and pathogen‐repelling coatings for textiles and provide valuable insights into the role of FLG in contact with Ag‐decorated Cu surfaces, which have the potential to revolutionize the development of high‐performance coatings for other surfaces.

## 2 Results and Discussion

### 2.1 Evaluation and Characteristic Properties of Antipathogen Materials

To create a platform that integrates both passive and active antipathogen properties, the critical components of the coating were thoroughly analyzed. It is evident that the flat surface of the microflakes, which are a few micrometers in diameter, is covered by small (> 50 nm) Ag grains (Figure S1, Supporting Information). The presented Ag‐decorated flakes are normally used as conductive paths on printed circuit boards. Both Cu and Ag utilize high pathogen activity in addition to low microbial resistance formation. ROS are normally formed upon oxidative dissolution, usage of nanoparticles or electrolytic interaction between the more and less noble metals. Furthermore, Meister et al.^[^ ^26^ ^]^ revealed that co‐deposited Cu on Ag or Ag on Pt offers higher antibacterial protection in contrast to pure Ag as sacrificial anode surfaces due to the electrochemically driven enhanced dissolution of Ag ions. Alongside the aforementioned strategy, we observed that the presence of edge imperfections in graphene structures frequently leads to the oxidation of the underlying copper, despite graphene's reputation as a highly effective anti‐corrosion material. The corrosion enhancement effect of graphene was attributed to its ability to promote electrochemical corrosion of the substrate near the edge.^[^ ^27^, ^28^, ^29^ ^]^

**Figure 1:**
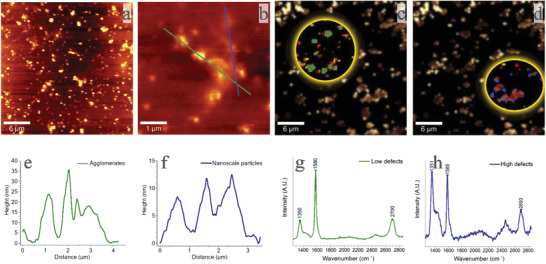
AFM ((30×30 microns a) and 5×5 microns b) image of FLG on a silicon wafer with the corresponding height profile e and f)) and Raman mapping ((30×30 microns) area with the presentation of a low defect area in green c) and its corresponding Raman spectrum g) and of a high defect area in blue d) with its corresponding spectrum h).

In the case of antipathogen action, the promotion of Cu oxidation is desired to achieve high protection with the lowest possible amount of Cu in the coating (Note 1, Supporting Information). It is hypothesized that FLG is a suitable material for such applications due to its crystal cleavage behavior of mechanically exfoliated sheets, which results in high reactivity at the edges. Edges are affected by the ribbon width and the orientations of C atoms at their edges. These C atoms present higher chemical reactivity due to unpaired electrons on their edges, offering a possibility for chemical modification of their edges,^[^ ^30^ ^]^ or they can be responsible for the oxidation of transition metals such as Cu. The FLG used in the experiments was prepared by mechanical high‐pressure liquid exfoliation of graphite. We studied the structural properties of FLG used in our experiments by Raman spectroscopy and AFM. For Raman analysis, a highly diluted FLG water dispersion (0.1% w/w) was applied to a glass wafer, and for AFM, a highly diluted FLG water dispersion (1% w/w) was applied to a silicon wafer by the drop cast method. The sample was then exposed to a high vacuum and a temperature of 200°C overnight for the elimination of surfactant. AFM is commonly used to determine the thickness of 2D carbon materials and related 2D materials. As shown in **Figure** **1**a, FLG sheets spread evenly on the surface of the silicon wafer. Due to the difference in height and substrate of the area covered by the FLG sheet, the morphology and height information of the sample can be observed from the 2D image. The FLG sheet agglomerates have a measured thickness ranging from 5 to 20 nm (Figure 1b, FLG agglomerates (Figure 1e) and FLG nano (Figure 1f). For the determination of FLG nano, we chose to test a region where the FLG sheets are relatively independent.

To facilitate the study of the oxidation effects of FLG on Cu, FLG islands were evaluated by Raman mapping, where the absorption intensity ratios of the D, G and 2D bands were emphasized for the determination of high‐ and low‐defect regions on FLG islands. On the 30×30‐micron mapping image (Figure 1c,d), we present the distribution of two different FLG species marked by two circles according to the D/G ratio. Particles highlighted in green (Figure 1c), mainly in the center of FLG islands, reveal low defect regions according to the average Raman spectrum (Figure 1g). The D band (1350 cm^−1^) intensity is very low in contrast to the intense sharp G band at 1580 cm^−1^. In comparison, the particles highlighted in blue (Figure 1d), mainly along the edges of the FLG islands, reveal the presence of high defect areas according to the average Raman spectrum (Figure 1h), seen as an intense D band (1530 cm^−1^) with a shoulder at 1404 cm^−1^ and a G band at 1585 cm^−1^. It should be mentioned that average spectra are calculated from all relevant data in the entire mapping area, resulting in a few hundred pixels with hundreds of spectra for both high and low defect areas; therefore, we cannot expect an exact match with a single spectrum measured on a defined surface where only a few layers are presented.^[^ ^31^ ^]^ Furthermore, our goal was not to evaluate FLG in detail, such as the number of layers or type of ribbon, but just to confirm the presence of disorder/defects on the edge through the D/G ratio of our material used in the experiments, resulting in higher antipathogen activity when used in combination with Cu flakes.

### 2.2 Morphological and Coating Characteristics

**Figure 2:**
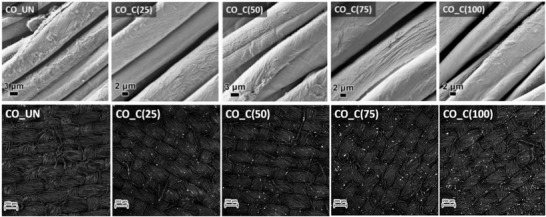
Scanning electron microscope (SEM) images of the studied untreated and treated cotton samples. (Top row) and Back scatter electron (BSE) images of the samples functionalized with coatings with varying concentrations of biocidal components (bottom row).

After a detailed evaluation of the materials used in the antipathogen service, we prepared the coatings on cotton textile. The presented coatings, containing either only one of the biocidal components (CuMF or FLG) or their combination at different concentrations, were prepared and applied to the cotton samples using the pad‐dry‐cure process, where minimal influence on fiber morphology is expected (details in the Experimental section). The morphology of the untreated cotton samples, the cotton samples treated with coatings containing only one component (CO_C‐CuMF and CO_C‐FLG) or the cotton samples treated with coatings containing both components at different concentrations (mg/mL) (CO_C(25), CO_C(50), CO_C(75) and CO_C(100)) were examined using SEM. The images are presented in **Figure** **2**.

The SEM microphotographs show the uneven morphology of the untreated cotton fibers; cellulose fibrils are clearly visible. After applying the polyurethane (PU) coating containing only CuMF (CO_C‐CuMF) to the cotton fibers, the fiber surface appeared to be smooth, indicating that the PU coating uniformly covered the fibers and that the CuMF was embedded within its matrix. On the other hand, the coating on the surface of the CO_C‐FLG sample was more textured, which is attributed to the presence of the FLG sheets in the matrix. The samples with coatings containing CuMF/FLG composites exhibited similar morphologies. As the concentrations of the two biocidal components in the coating applied to the cotton fibers increased, the morphology of the coating on the fibers increased in texture. Overall, the coatings were homogeneously distributed over the entire surface of the fibers, while the PU matrix did not cause the fibers to adhere to each other, indicating that the coating layer on the fibers was extremely thin.

Next, SEM/BSE analysis of the samples treated with both biocidal components at different concentrations was performed to compare the concentrations and locations of CuMF on the surfaces of the fibrous samples (Figure 2).

The SEM/BSE images show a contrast between the organic fibers (dark areas indicating low z elements) and the highlighted inorganic CuMF (bright spots on the fibers indicating high z elements). Thus, the results reveal an important difference between the samples, namely, that the amount of CuMF on the fibers increased as the concentration of biocidal components in the coating increased. Moreover, it is evident from the images that the flakes were randomly distributed and did not form larger clusters or agglomerates, regardless of the concentration of the flakes in the coating applied to the fiber surface.

**Figure 3:**
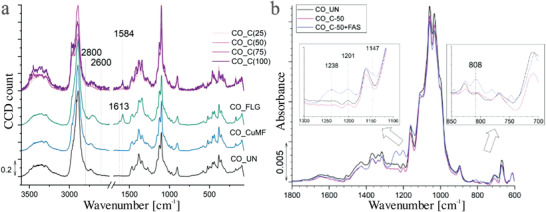
Raman spectra in the region at 3600–70 cm^−1^ of the studied samples a) and FT‐IR spectra of the CO_UN, CO_C(50) and CO_C(50)+FAS samples in the region of 1800–600 cm^−1^ b).

The presence of FLG on the functionalized fibers could not be detected by the previously used methods since carbon is also present in the cotton fibers themselves. Therefore, Raman analysis was used to detect FLG on the fiber surface (**Figure** **3**a). The spectra of all the samples show characteristic cellulose peaks. The peak at 2896 cm^−1^ belongs to the asymmetric and symmetric vibrations of CH_2_, while the CH_2_ deformation vibration peaks are observed in the 1470–1345 cm^−1^ region; however, the peak at 1345 cm^−1^ could also represent the deformation vibrations of OH groups. The stretching vibrations of the β−1,4‐glycosidic bonds between the glucose units are expressed as peaks in the region of 1150–1100 cm^−1^, while the 1150 cm^−1^ peak also represents the asymmetric stretching vibrations of the C─C ring. The symmetric stretching vibration of the C─O─C bond appears at 1126 cm^−1^, and the asymmetric stretching vibration of this bond is shown as a peak at 1100 cm^−1^. Furthermore, the C–C–O ring deformation appears as peaks at 463 and 436 cm^−1^, and the C–C–C ring deformation vibrations appear at 382 cm^−1^.^[^ ^32^ ^]^

In addition to the peaks already mentioned, the CO_FLG sample shows FLG peaks, indicating its presence in the coating. Namely, the G‐band, which is universal in sp2 carbon systems and represents the stretching of the C─C bond, is observed at 1584 cm^−1^.^[^ ^33^, ^34^ ^]^ Moreover, the peak at 1613 cm^−1^ represents the D’ peak,^[^ ^33^ ^]^ while the 2D peak between 2600 and 2800 cm^−1^ could not be clearly distinguished due to the cellulose peaks in this region.^[^ ^33^ ^]^ In the case of the samples functionalized with coatings containing different concentrations of FLG and CuMF, a peak at 1584 cm^−1^ also appeared, although its signal was lower for the CO_C(25) and CO_C(50) samples due to the lower FLG concentrations in the coating formulation. However, the CO_C(75) and CO_C(100) samples showed obvious peaks, indicating successful incorporation of FLG into the coating matrix.

The quest for self‐sanitizing textiles with unparalleled antibacterial and antiviral protection requires not only active properties, but also passive protection to effectively combat pathogens. To physically prevent the transmission of respiratory droplets, it is crucial to have a low surface energy of the sample material. This combination of active and passive defense mechanisms creates a comprehensive approach to provide superior protection against bacterial and viral threats. By incorporating a low surface energy into self‐sanitizing textiles, we can take a significant step toward creating a safer and healthier environment. For this reason, the CO C(50) sample was additionally coated with the perfluoroalkyl‐polysiloxane FAS matrix. The morphological and chemical properties of the CO_C(50)+FAS sample were further investigated by SEM, EDS spectroscopy, EDS mapping and FTIR. Compared to the surface morphology of the CO_C(50) sample, the surface of the CO_C(50)+FAS sample (Figure S2, Supporting Information) appears rougher due to the presence of the additional hydrophobic coating component. The FTIR spectra (Figure 3b) show the cellulose fingerprint in the region of 1500–800 cm^−1^, which represents the vibrations of the C─H, C─C, C─O, C─O─C and O─H bonds in the glucoside rings. Furthermore, the peak at 1645 cm^−1^ represents the O─H bending of the absorbed water.^[^ ^35^, ^36^, ^37^ ^]^ While the CO_UN and CO_C(50) samples show similar spectral characters, the presence of the perfluoroalkyl‐polysiloxane FAS matrix can be clearly observed on the CO_C(50)+FAS sample in the form of three peaks, at 1238, 1201, and 1147 cm^−1^ belonging to a mixture of ν_a_ and CF_2_ group rocking, ν_a_ of CF_2_ and CF_3_, and ν_s_ of CF_2_ modes, respectively. Moreover, the symmetric stretching vibrations of Si‐O‐Si groups can be observed from the peak at 808 cm^−1^.^[^ ^35^, ^38^ ^]^

It can be inferred from the analysis of the morphological and chemical properties of the samples that the applied coatings were evenly spread over the functionalized fibers and that both functional components were integrated within the coatings on the functionalized fibers.

### 2.3 Coating Surface Interaction with Bacteria or Virus

**Figure 4:**
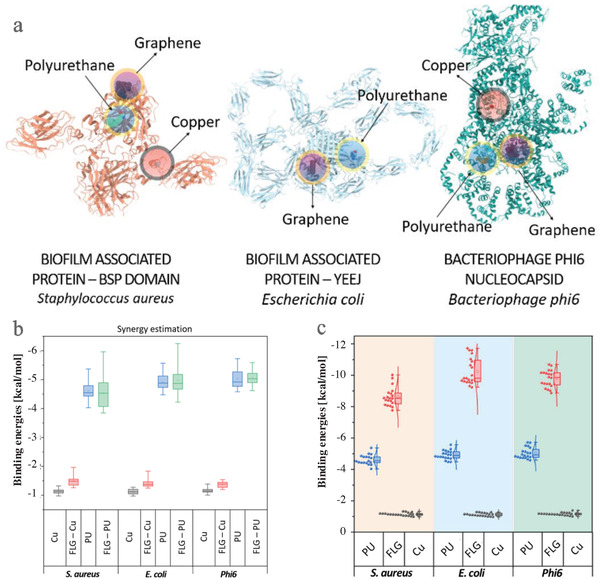
Molecular interaction a), Synergy estimation b) of graphene, copper and polyurethane with gram‐positive (*S. aureus*), gram‐negative (*E. coli*) and bacteriophage, elucidating their antibacterial and antiviral mechanisms and the interaction analysis results c) for polyurethane (PU), graphene (FLG) and copper (Cu) and presentation of biofilm‐associated proteins.

To understand the molecular mechanism behind the antibacterial properties, an in‐silico analysis was conducted, revealing strong interactions with both types of bacteria at distinct protein catalytic sites and with various amino acids. **Figure** **4**a illustrates the specific domains on *S. aureus, E. Coli* and *Bacteriophage phi6* that are responsible for their interaction with the anti‐pathogen coating components. Additionally, the strength of the interaction was evaluated by determining the binding energy (Figure 4b) of the active sites in relation to the coating components. The bonding energy of the interaction with graphene was found to be greater for *E. coli* compared to *S. aureus*. Similarly, a slightly higher bonding energy was observed between polyurethane binder and *E. coli* compared to *S. aureus*. It's noteworthy that the strong interaction of the PU binder with active sites on the tested living species is desirable, as only a strong interaction can lead to effective antipathogen protection. The results of the interaction analysis were consistent with the findings from the experiments. Furthermore, the estimation of the synergistic impact utilized a graphene complex as a foundation. The findings shown in Figure 4c suggest that the combination of graphene, polyurethane, and Cu exhibit robust binding to both types of bacteria. The reason for this synergistic antibacterial effect is due to the combined biocidal power of both components and graphene's ability to effectively receive electrons.

### 2.4 Evaluation of Antipathogen Properties

To evaluate the protective properties of the samples, their antibacterial and antiviral activities were determined. First, the antibacterial properties of the samples were investigated by determining the reduction of *E. Coli* and *S. aureus* upon their contact with the samples (**Table** **1**).

**Table 1:** Antibacterial activity of the samples against *E. coli* and *S. aureus*, shown as bacterial colonies grown on agar plates inoculated with a bacterial suspension shaken with the studied samples for 2 h, and the log reduction values, calculated from the difference in CFUs of treated and untreated samples.

Sample	Bacterial growth		Log reduction	
	*E. coli*	*S. aureus*	*E. coli*	*S. aureus*
CO_UN	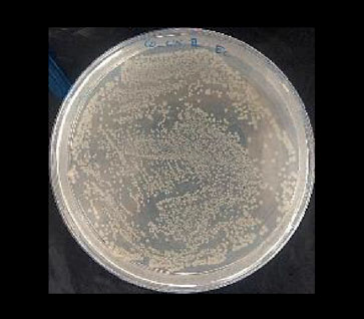	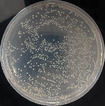	/	/
CO_C‐CuMF	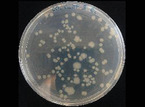	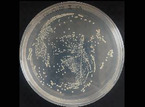	1.01	0.25
CO_C‐FLG	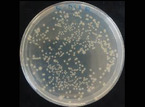	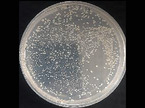	0.91	0.12
CO_C(25)	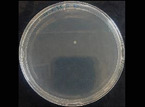	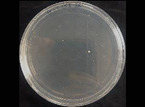	3.24	3.12
CO_C(50)	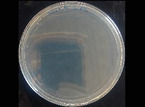	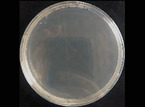	9.81	3.17
CO_C(75)	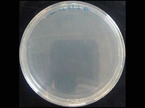	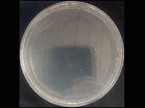	9.81	3.22
CO_C(100)	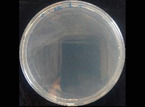	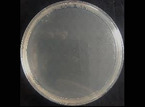	9.81	3.47

When both biocidal components were applied to the samples, either separately or combined, the samples showed higher overall efficiency in reducing gram‐negative bacteria *E. coli* compared to gram‐positive bacteria *S. aureus*. The outer layer of the bacterial cell is the first contact point with the biocidal compound, which is why the antibacterial efficiency of the compounds is different for the two types of bacteria. The outer layer of gram‐negative bacteria consists of a thin outer membrane, whereas the outer layer of gram‐positive bacteria consists of a thick layer of peptidoglycan. In the case of graphene material, different antibacterial action toward the two different types of bacteria has been found by Pulingam et al.^[^ ^39^ ^]^ When he dispersed graphene oxide sheets in media containing bacteria, a wrapping antibacterial mechanism was observed in the case of *S. aureus*, as the peptidoglycan layer interacted with the graphene sheets. On the other hand, in the case of *E. coli*, damage to the membrane occurred due to the sharp edges of the graphene sheets, resulting in a decrease in intracellular density. In our case when only FLG is embedded in the PU coating, the wrapping mechanism was obstructed as the FLG sheets were bound in the coating, which could explain the lower antibacterial activity of this sample toward gram‐positive bacteria. Furthermore, the more efficient antibacterial activity of Cu particles against gram‐negative bacteria has also been shown in the literature, particularly in the study of Sathiyavimal et al.^[^ ^40^ ^]^ There, this activity was attributed to the thickness of the outer layers of the bacterial cells since the peptidoglycan layer of gram‐positive bacteria is wider than the cell wall of gram‐negative bacteria. We hypothesise that optimal ROS generation out of flakes and FLG was responsible for outstanding biocidal action (Note 2, Supporting Information).

Furthermore, when the coating containing only one biocidal component CuMF or FLG was applied, poor antibacterial activity was obtained with a log reduction of 1 or less against both types of bacteria, indicating insufficient biocidal properties. In contrast, the samples with the two‐component coatings resulted in a high log reduction of more than 3 (i.e., >99.9% growth reduction) of both bacteria, regardless of the concentration of the biocidal components within the coating applied to the textile fibers. The synergy was also investigated by in silico analysis.

Thus, the results clearly demonstrate the synergistic effect of CuMF and FLG: in the case of gram‐positive *S. aureus*, an excellent reduction in the region of 3.12 to 3.47 log was achieved, while in the case of gram‐negative *E. coli*, the CO_C(50), CO_C(75) and CO_C(100) samples exhibited an extraordinary reduction, with a log value of 9.81 recorded.

In fact, the proposed antibacterial action of Cu occurs through the release of dissolved Cu ions (generation of ROS), which damage the membranes and outer envelopes of bacteria via oxidation of the proteins and lipids.^[^ ^41^, ^42^, ^43^ ^]^ Following degradation, the released Cu ions can enter and accumulate in the bacterial cell. This leads to cell death as a result of oxidative stress, which damages the proteins, nucleic acids and lipids.^[^ ^14^, ^41^, ^44^, ^45^ ^]^ However, as mentioned earlier, FLG is also a good electron acceptor; therefore, it interacts with the negatively charged envelopes, contributing to greater damage to lipid membranes.^[^ ^46^, ^47^ ^]^ In this way, access to Cu ions that can enter the bacterial cell is boosted, leading to superior biocidal activity. Since the optimal concentration of biocidal components in the coatings denotes the minimal concentration required to achieve excellent antibacterial protective properties, the CO_C(50) sample was selected for further investigation because it provided a log reduction of 3.17 and 9.81 for *S. aureus* and *E. coli*, respectively. This sample was also tested for its antiviral activity, and the results are presented in **Table** **2**.

**Table 2:** Reduction of bacteriophage phi6 in the presence of the CO_CuMF, CO_FLG and CO_C(50) samples.

Sample	Phi6 reduction	
	[%]	log
CO_CuMF	99.98	3.75
CO_FLG	99.05	2.02
CO_C(50)	>99.99	5.53

To study the antiviral activity of the studied samples, bacteriophage phi 6 was chosen. This bacteriophage has already been used as a model for coronaviruses and influenza viruses, as it is an enveloped virus that is extensively characterized, safe to handle, and allows accurate and rapid quantification of virus infectivity using plaque assays.^[^ ^48^ ^]^ Analogous to the results obtained from the antibacterial tests, the contact of phi6 with the CO_CuMF sample resulted in a higher reduction of the virus compared to the CO_FLG sample. Furthermore, the two biocidal components incorporated into the coating of the CO_C(50) sample exhibited synergistic antiviral behavior and showcased an excellent reduction of phi6, with a log value of 5.53, which is in the range of excellent according to ISO 18 184:2019.

It needs to be emphasized that the concentration of the biocidal components within this sample (CO_C(50)) was only half of the concentration of the reference samples containing only one of both biocidal components. The results were in line with the in‐silico analysis of the interaction study of materials with vial membrane proteins, where they were found to interact firmly with amino acids of viral proteins via strong H‐bonds (Figure 4). The synergistic antiviral activity results of copper and FLG recorded here are consistent with those of a previous study,^[^ ^11^ ^]^ where Cu_2_O nanoparticles and single sheet graphene were used as antiviral coating components and tested against an influenza A reporter virus. In their work, the proposed synergetic mechanism between the two components was attributed to the interaction of graphene with the viral lipid bilayer membrane, resulting in an aggregation of virus particles on the FLG sheets. This ensured better contact of the virus with the Cu particles, which hindered the structure and function of the HA protein responsible for viral entry into the host cell, thus preventing cell infection. It is highly likely that similar mechanisms of inactivation also took place in our work, with the FLG interacting with the viral envelope, while the Cu‐affected protein P3 was responsible for the attachment of phi6 to the bacterial pilus and, hence, bacterial infection.^[^ ^49^ ^]^

**Figure 5:**
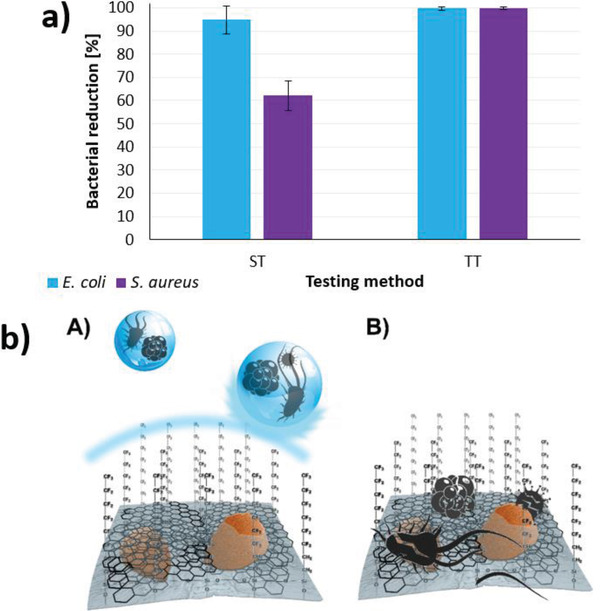
Bacterial reduction of *E. coli* and *S. aureus* for the CO_C(50)+FAS sample determined by the shaking test (ST) and the transfer test (TT) a); a schematic illustration of the double antibacterial and antiviral action of the CO_C(50)+FAS sample b), representing passive protection due to the low surface energy of the sample provided by FAS (A) and active protection due to the synergistic antibacterial and antiviral action of CuMF and FLG (B).

It has been established that for bacteria to colonize and form biofilm on fabric surfaces, their initial adherence to the fiber surface is crucial and is directly linked to the fiber's surface free energy. This can be effectively reduced by creating a low surface energy of the fibers (Figure S3 and Note 3, Supporting Information). Therefore, to investigate the passive antibacterial activity of the CO_C(50)+FAS sample, the transfer test was used together with the shaking test. The results are presented in **Figure** **5**a. The antibacterial activity of the CO_C(50)+FAS sample determined by the shaking test was quite good but still much worse than that of the CO_C(50) sample without FAS. Based on the experience from previous studies,^[^ ^48^ ^]^ the reason for this is the lack of close contact between the bacterial inoculum and the surface of the fibers due to their hydrophobic nature, which prevents complete wetting of the CO_C(50)+FAS sample. In contrast, the results of the transfer test, which ensures good contact of the sample with the bacterial inoculum by pressing the sample against the inoculated agar surface, showed complete, i.e., >99% reduction of both bacteria tested. Minimal influence on cytotoxicity is present (Note 4, Supporting Information).

This clearly indicates that the CO_C(50)+FAS sample possesses both passive and active antibacterial activity, i.e., protection against bacterial adherence to the fiber surface and synergistic antibacterial action of the CuMF/FLG complex, which would ensure immediate destruction of the bacteria if they are still somehow able to adhere to the fibers despite the reduced surface free energy of the fibers.

It should also be noted that the antiviral activity of the CO_C(50)+FAS sample could not be determined because the protocol for determining the antiviral activity of a hydrophobic sample has not yet been established. Based on the results of the shaking test, the presence of FAS would also disable the complete wetting of the sample in this case, which is a prerequisite for the adequate performance of the test for antiviral activity. Nevertheless, passive antiviral activity is also expected, analogous to bacterial activity. The two protective mechanisms are shown schematically in Figure 5b.

## 3 Conclusion

The newly developed PU textile coating (Figure S5, Supporting Information) with the addition of CuMF/FLG composite has demonstrated exceptional efficacy in providing biocidal protection against pathogens. The coating operates with a dual active and passive antipathogen action and the biocidal components CuMF and FLG show strong reductions of both gram‐positive and gram‐negative bacteria at low concentrations. The CO_C(50) sample, with optimal properties, was found to have high antiviral properties and to significantly reduce *S. aureus* and *E. coli*. The incorporation of low surface energy carrier (FAS) led to a water contact angle of 144°, providing both passive protection through low surface energy and active self‐sanitizing antibacterial properties. It is worth highlighting that our work introduces a comprehensive methodology to gain knowledge on the industrial approach to produce antipathogen coatings from raw materials available on the market, which is generally lacking and could be efficiently used to guide future developments of coatings for industrial applications (Note 5, Supporting Information). Moreover, such coatings could be applied to various textile substrates for personal protective equipment and has the potential beyond textile for broader applications.

## 4 Experimental Section

### Materials

Alkaline‐scoured, bleached, and mercerized 100% cotton fabric with 125 g m^−2^ mass area (warp density: 38 threads/cm; weft density: 28 threads/cm) was purchased from Tekstina d.d., Slovenia. For the coating preparation, 2‐propanol (Merck KGaA, Germany), glycerol (Sigma–Aldrich, USA), wetting and dispersing additive Disperbyk 190 (BYK‐Chemie GmbH, Germany), water‐based aliphatic PU dispersion Alberdingk U 900 (Alberdingk Boley GmbH, Germany), and aqueous dispersion of colloidal silica Levasil cc 301 (Nouryon, The Netherlands) were used. As biocidal components, CuMF (eConduckt Copper 04 2500) was purchased from Eckart, Germany, and FLG suspension was provided by Cami Consultancy LTD, England. As a water‐repellent agent, the fluoroalkyl functional water‐borne siloxane (FAS) Protectosil F 8815 (Chemcolour, Slovenia) was used.

### Preparation of the Coatings

Mixtures containing CuMF and FLG were prepared separately in two beakers. Mixture 1 contained 16.6 g of 2‐propanol, 1.66 g of glycerol, an appropriate amount of CuMF (listed in **Table** **3**) and one drop of Disperbyk 210. The mixture was stirred with a magnetic stirrer for 10 min at 470 rpm. Mixture 2 (100 mL) contained 2 g of Disperbyk 190 and the appropriate amount of FLG suspension (listed in Table 3) in water. Mixtures 1 and 2 were then combined and mixed on a dispersing instrument (Ika Ultra Turrax T 25 easy clean digital) at 5000 rpm for 15 min. After mixing, 66.66 mL of 5% diluted PU dispersion Alberdingk U 900 and 5 g of Levasil cc 301 were added to prepare the coating bath, which was mixed for an additional 10 min using a magnetic stirrer. The pH of the coating bath was determined and set to 8. Two coating baths containing only one biocidal component (CuMF or FLG) were also prepared. The coating bath codes and amounts of biocidal components are presented in Table 3.

**Table 3:** Amounts of biocidal components added in coating baths and the corresponding coating bath codes.

Coating bath code	Concentration of biocidal components	
	CuMF [g]	FLG (dry mass) [g]
PU/CuMF	0.660	/
PU/FLG	/	1.00
PU/CuMF+FLG(25)	0.165	0.25
PU/CuMF+FLG(50)	0.330	0.50
PU/CuMF+FLG(75)	0.495	0.75
PU/CuMF+FLG(100)	0.660	1.00

### Functional Finishing of Cotton Fabric

**Figure 6:**
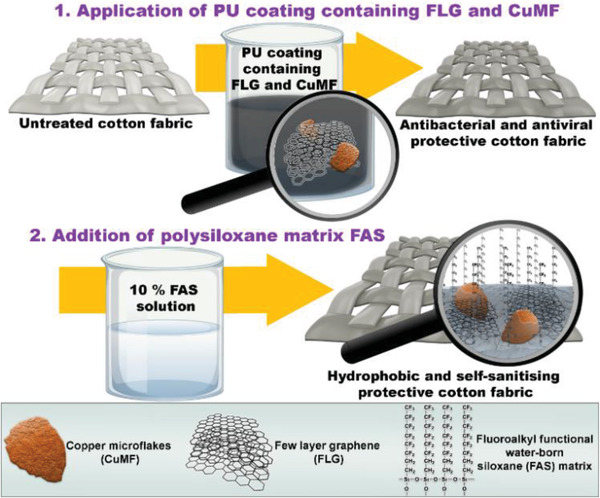
Schematic presentation of the cotton functionalization process.

The prepared functional coatings were applied onto the cotton fabric samples using the pad‐dry‐cure method, which involved immersing the fabric into the coating bath, followed by wringing with a wet pick‐up of 80±5%, air drying, and curing at 70 °C for 10 min. FAS was also applied by the pad‐dry‐cure method, where the sample was fully immersed into 10% FAS precursor solution (wet pick‐up 80±5%), followed by 5 min drying at 80 °C and 5 min curing at 150 °C. The sample codes, with descriptions of their functionalization, were presented in **Table** **4**, and the functionalization process is shown in **Figure** **6**.

**Table 4:** Sample codes according to the chemical modification of the cotton fabric.

Sample code	Description of chemical modification
**CO_UN**	No chemical modification
**CO_C‐CuMF**	Cotton fabric finished with PU/CuMF coating bath
**CO_C‐FLG**	Cotton fabric finished with PU/FLG coating bath
**CO_C(25)**	Cotton fabric finished with PU/CuMF+FLG(25) coating bath
**CO_C(50)**	Cotton fabric finished with PU/CuMF+FLG(50) coating bath
**CO_C(75)**	Cotton fabric finished with PU/CuMF+FLG(75) coating bath
**CO_C(100)**	Cotton fabric finished with PU/CuMF+FLG(100) coating bath
**CO_C(50)+FAS**	Cotton sample CO_C(50) additionally finished with FAS

### Analysis and Measurements

*Scanning electron microscopy (SEM) and energy‐dispersive X‐ray spectroscopy (EDS)*: The morphology of the samples was determined by scanning electron microscopy at an accelerating voltage of 1 kV for SEM or 15 kV for EDS using an FE‐SEM Zeiss SUPRA 35 VP instrument (Oberkochen, Germany) equipped with an energy dispersive spectroscopy (EDS) solid state detector (SDD) (Oxford Instruments, High Wycombe, UK). Prior to observation, the samples were coated with carbon to ensure sufficient electrical conductivity and to avoid charging effects. SEM micrographs were recorded using both secondary electron (SE) and backscattered electron (BSE) imaging modes. BSE compositional (Z‐contrast) imaging was applied to emphasize and expose the difference between the CuMF and the cotton fiber.

### Raman Spectroscopy

A WITec Alpha 300AR confocal Raman microscope was used for Raman spectra and AFM measurements. For the analysis, a 50x objective and laser wavelength of 532 nm with a laser power of 15 mW were used for all of the samples, with the exception of CO_C‐CuMF, where 1 mW laser power was used.

### Fourier Transform Infrared Spectroscopy

The Fourier transform infrared (FTIR) spectra of the prepared materials were obtained on a Bruker instrument (Vertex 70v, Germany). The spectra were recorded over the region of 4000–600 cm^−1^ with a resolution of 4 cm^−1^ and averaged over 32 spectra.

### Antibacterial Activity

The antibacterial activity of the studied cotton samples was estimated against the pathogenic bacteria gram‐negative Escherichia coli (ATCC 25 922) and gram‐positive Staphylococcus aureus (ATCC 6538).

The bacterial reduction was determined according to two standard methods. The ASTM E 2149−01 standard method (shaking test) was performed on all of the prepared samples by inoculating 1 g of the sample with 20 mL of a bacterial suspension of 105 CFU/mL in a flask, which was then shaken using a wrist‐action shaker for 2 h. Afterward, 40 µL of each suspension was spread on nutrient agar and incubated for 24 h at 37 °C. The second method, EN ISO 20 743:2007 (transfer test), was used on the CO_C(50)+FAS sample. A bacterial suspension of 105 CFU mL^−1^ was prepared, and 1 mL of the suspension was spread on each agar plate. Each parallel sample was then pressed onto the inoculated agar for 1 min using a 200 g weight. The pressed samples were incubated for 24 h at 37 °C in a humid environment. Afterward, the samples were placed in a sterile flask, and 20 mL of Milli‐Q water was added. Each of the samples was shaken on a vortex shaker for 1 min, and 40 µL of the suspension was spread on agar and incubated at 37 °C for 24 h. For both methods, the reduction of bacterial growth, R (Equation 1), and antibacterial activity, A (Equation 2), were calculated as follows:

(1) $$R\left(\%\right)=\frac{U-T}{U}$$

(2) $$A\left(\log\right)=\log\left(U\right)-\log\left(T\right)$$

where U is the number of colony‐forming units (CFU) on the untreated sample after incubation for 2 h, T is the number of CFUs on the treated sample grown on agar plates under the same conditions, log U is the average common logarithm for the number of bacteria obtained from the control (untreated) sample after shaking for 2 h, and log T is the common logarithm for the number of bacteria obtained from the treated sample after shaking for 2 h. The values were calculated from the numbers adjusted to the number of CFUs per 1 mL. The R and A values represent an average of 3 parallels and 4 inoculated agar plates per parallel, resulting in 12 total CFU counts per sample.

### Antiviral Activity

To determine the antiviral activity values of the cotton samples, (DSM 21 518) was used, and a modified protocol from standard ISO 18 184:2019 was followed. Cotton samples (CU_UN, CO_C‐CuMF, CO_C‐FLG and CO_C(50)) were cut into 20×20 mm pieces with each having a mass of 0.40 g ± 0.05 g. They were placed in 50 mL falcon tubes and inoculated with 0.2 mL of virus solution at a concentration of 3–5×105 plaque‐forming units (PFU)/mL. The inoculated cotton samples were washed with 20 mL of SCDLP medium and vortexed either immediately after inoculation (CU_UN, Va) or after 2 h of incubation at room temperature (CU_UN, Vb; CO_C‐CuMF, CO_C‐FLG or CO_C(50), Vc). The appropriate dilutions of virus samples were then prepared, and virus concentrations were determined using a double‐layer plaque assay (see below). Antiviral activity was calculated as follows:

(3) $$Mv\left(\%\right)=\frac{Va-Vc}{Va}$$

(4) $$Mv\left(\log\right)=\log\left(Va\right)-\log\left(Vc\right)$$

where Mv is the antiviral activity value and Va is the average virus concentration determined immediately after the inoculation of CU_UN, whereas Vc is the average virus concentration of samples CO_C(50), CO_C‐CuMF or CO_C‐FLG after 2 h of incubation. The V values represent an average of 3 parallels per virus dilution, resulting in up to 12 total PFU counts per sample. Samples CO_C(50) and CU_UN were tested in three independent repetitions. To verify the adequacy of the test, the M value was also calculated according to Equation 5:

(5) $$M=\log\left(Va\right)-\log\left(Vb\right)$$

where M is the reduction value, Va is the average virus concentration immediately after the inoculation of CU_UN, and Vb is the average virus concentration of CU_UN after 2 h of incubation. M values should not exceed log 1.

### Double‐Layer Plaque Assay

Double‐layer plaque assays (DALs) were performed to determine the concentration of viruses after exposure to cotton fabric with or without antimicrobial components. The following three types of media were used: “TSB agar” for the agar plates, “TSB top agar” for the top layer, and “liquid TSB” for culturing the bacterial host strain Pseudomonas syringae van Hall 1902 (DSM 21 482). “TSB agar” was prepared from 30 g L^−1^ BD tryptic soy broth (TSB) and 15 g L^−1^ Bacto agar. “TSB top agar” was prepared in the same manner, except with the addition of 7 g of agar instead of 15 g of agar. “Liquid TSB” was prepared from 30 g of TSB/L. All media contained 1.93 g L^−1^ of MgCl_2_ ×6H_2_O. A bacterial host culture was used in the log phase, which was prepared by inoculating 0.2 mL of at least 2‐day‐old bacterial culture into 5 mL of liquid TSB medium, followed by ≥3 h of incubation (25 °C, 200 rpm). DAL was carried out by adding 0.2 mL of bacterial host and 0.25 mL of undiluted or diluted virus samples to ≈5 mL of melted “TSB top agar” in 15 mL glass tubes. The mixture was thoroughly mixed and poured onto agar plates in three replicates. The plates were incubated overnight at 25 °C, after which the number of plaques was counted. Virus concentrations were determined by considering virus dilutions and plating volumes and were given as the average (three technical replications) PFU/mL (Note 6, Supporting Information).

### Contact Angle Measurements

The static contact angle measurements were carried out with water on the fabric samples using a DSA 100 contact angle goniometer (Krüss, Germany). The static contact angle, θ, of a liquid droplet was determined using the Young–Laplace fitting method. Five measurements, which were performed with 5‐µL liquid droplets placed at different points on the fabric samples, were used to calculate the average contact angle values and standard errors. All values reported here correspond to contact angles obtained under stationary conditions, i.e., 30 s after the liquid droplet was placed on the fabric.

### In Silico Analysis

The molecular analysis of the antibacterial effect of the compounds was performed using a computational approach by molecular docking. Graphene, copper oxide and polyurathrene were studied for their interaction with bacterial membrane proteins and proteins associated with biofilm formation. To determine their effects on the bacterial surface, different proteins were selected for different bacterial species. For example, biofilm‐associated proteins from BSP domains were selected for *S. aureus*; similarly, YEEJ proteins were selected for *E. coli*, which plays an important role in the formation of biofilms. PHI6 proteins were taken for interaction in the case of bacteriophage, as they were present on the surface. GO, copper oxide and polyurethane structures were obtained from X‐ray methods that have been previously described. AutoDock 4.0 was used for the docking studies. The Lamarckian Genetic Algorithm was used with a population size of 150 and a maximum number of evaluations set to 2 500 000. To analyse the conformations and clustering after docking, AutoDock and Chimera were used.

## Conflict of Interest

The authors declare no conflict of interest.

## Author Contributions

I.J., S.B., D.Š. performed conceptualization; D.Š., K.F., N.V.de V., A.D., S.K.V., P.K.P. performed formal analysis; I.J., P.K., B.S. performed funding acquisition; D.Š., I.J., A.F., P.K. performed methodology; I.J. performed project administration; D.Š., I.J. performed roles and wrote the original draft; D.Š., A.D., P.K., A.F., B.S., B.T., S.B., S.K.V., P.K.P., I.J. wrote, reviewed and edited the manuscript.

## Supporting information

Supporting Information

## Data Availability

The data that support the findings of this study are available from the corresponding author upon reasonable request.
