# Supplementary material for: Boosting Copper Biocidal Activity by Silver Decoration and Few‐Layer Graphene in Coatings on Textile Fibers
Source: Glob Chall. 2023 Sep 13;7(10):2300113. doi: 10.1002/gch2.202300113 (PMC10566802; doi:10.1002/gch2.202300113)
Supplement: Supplementary file 1 — Supporting Information [file GCH2-7-2300113-s001.pdf]

Global Challenges
Open Access

Supporting Information

for *Global Challenges.*, DOI 10.1002/gch2.202300113

Boosting Copper Biocidal Activity by Silver Decoration and Few-Layer Graphene in Coatings on Textile Fibers

*Danaja Štular, Nigel Van de Velde, Ana Drinčić, Polona Kogovšek, Arijana Filipić, Katja Fric, Barbara Simončič, Brigita Tomšič, Raghuraj S. Chouhan, Sivasambu Bohm, Suresh Kr. Verma, Pritam Kumar Panda and Ivan Jerman**

Supporting Information

Title:

Boosting Copper Biocidal Activity by Silver Decoration and Few-Layer Graphene in Coatings on Textile Fibres

*Danaja Štular[1], Nigel Van de Velde[1], Ana Drinčić[1], Polona Kogovšek[2], Arijana Filipič[2], Katja Fric[2], Barbara Simončič[3], Brigita Tomšič[3], Raghuraj S. Chouhan[4], Sivasambu Bohm[5], Suresh Kr. Verma[6,7], Pritam Kumar Panda[6], Ivan Jerman[1]**

[1]National Institute of Chemistry, Hajdrihova 19, 1001 Ljubljana, Slovenia

[2]National Institute of Biology, Večna pot 111, 1000 Ljubljana, Slovenia

[3]University of Ljubljana, Faculty of Natural Sciences and Engineering, Aškerčeva 12, 1000 Ljubljana, Slovenia

[4]Institute "Jožef Stefan", Department of Environmental Sciences, Jamova 39, 1000 Ljubljana, Slovenia

[5]Imperial College London, South Kensington Campus London SW7 2AZ, UK

[6]Ångströmlaboratoriet Lägerhyddsv.1 Box 530, 75121, Uppsala, Sweden

[7]School of Biotechnology, KIIT University, Bhubaneswar, India,751024

*Correspondence: ivan.jerman@ki.si, tel. +386 1 4760 440

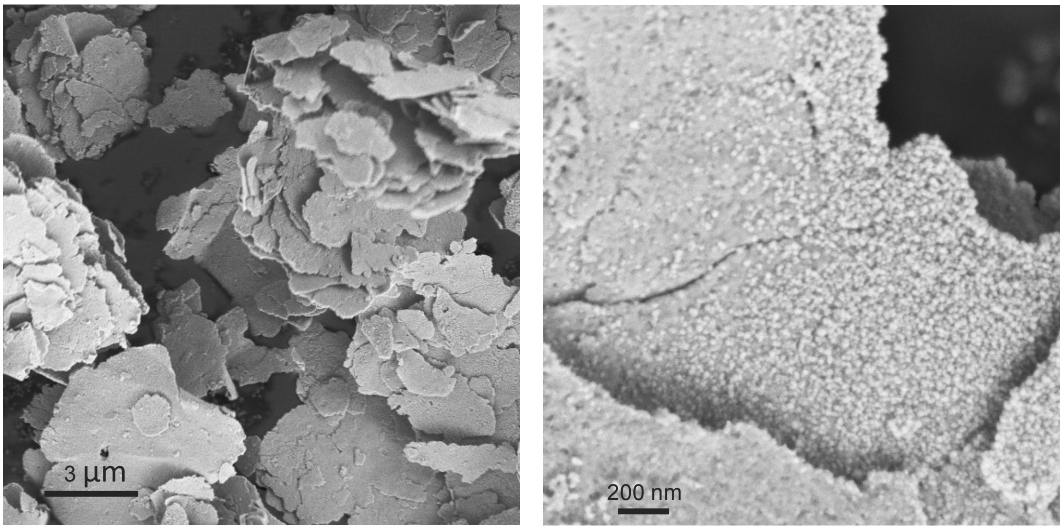

Figure S1. SEM images of silver decorated copper flakes used in research.

Supplementary Note 1 | Mechanism of releasing copper species

The release of ions from a solid surface can be tuned by the concentration of metal added in the coating, the particle size (smaller particles, more atoms on the surface-faster release) or the degradation of the binder (use of biodegradable polymer i.e. poly(DL-lactide-co-glycolide).[1]

Here, we use a material that has not been employed before for antibacterial/antiviral applications, namely silver-decorated copper flakes in combination with few-layer graphene (FLG).

To explain why copper and silver, which are commonly employed in scientific studies, are more effective at low concentrations when FLG is present, in this study we propose two unique concepts regulating the release of copper species. The first concept is reinforced by release kinetics based on an electrochemical sacrificial anode mechanism in which two materials (Cu/Ag) with different electropotentials are in contact. The addition of Ag (a more noble material) on the copper surface enables the concept of a Cu sacrificial anode electrode and increased release of Cu ionic species in contrast to using just pure Cu species. The second concept revolves around the interaction between the copper and the FLG's edge. It was confirmed by Raman analysis that the edges of the FLG used are full of defects. Disorders/defects on the edge, presented through the D/G bands ratio of our material, can cause the increased dissolution of copper ions. The release of metal cations leads to oxidative stress via the generation of reactive oxygen species (ROS).

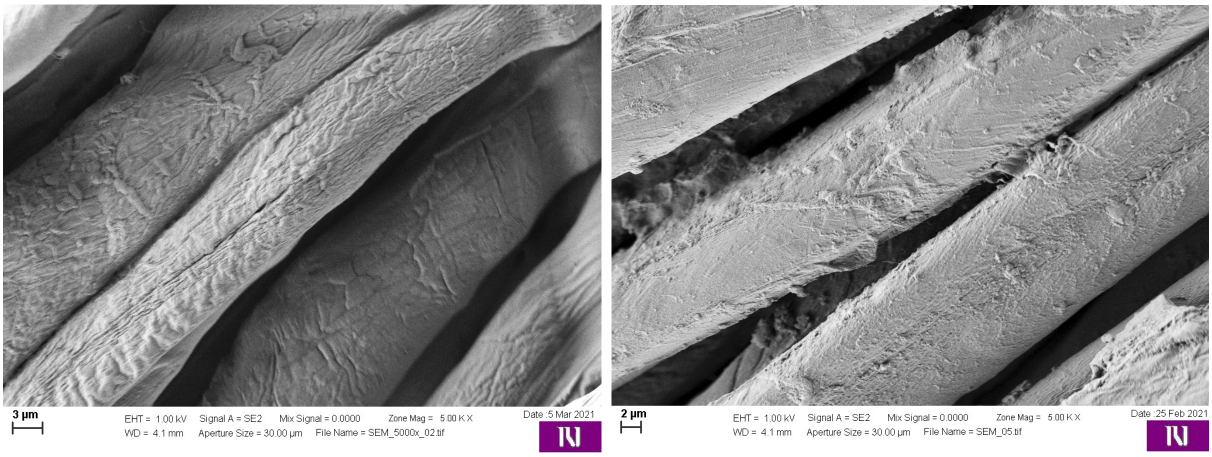

Figure S2. SEM image comparison of the surface morphology for the CO_C(50) sample (left) and the surface of the CO_C(50)+FAS sample (right).

---

[1]Ramakrishnan & Aprem. *Contraception*. **2015,** 92, (6) 585-8. 10.1016/j.contraception.2015.08.014

Supplementary Note 2 | Generation of Reactive Oxidation Species (ROS)

In the field of nanotechnology, it is generally accepted that a large number of inorganic nanomaterials (i.e. Ag, Cu) can facilitate intrinsic ROS generation[2]. Zhang et al.[3] gave a mini review on the mechanism of ROS generation. Liu et al.[4] tested a wide range of graphenic carbon materials (i.e. FLG) for their ability to generate ROS. Dissolved oxygen and water molecules are sufficient for the generation of ROS.[2, 4] It was shown that the reaction depends on the total surface area and is mediated by structural defects. Accordingly, in our study we also observed increased activity when a proper amount (sufficient amount of structural defects) of FLG was added to silver decorated flakes to build the anti-pathogen coating.

The results of our antimicrobial research demonstrate that the incorporation of FLG into a coating boosts its antipathogen action compared to coatings containing solely Ag-decorated Cu flakes.

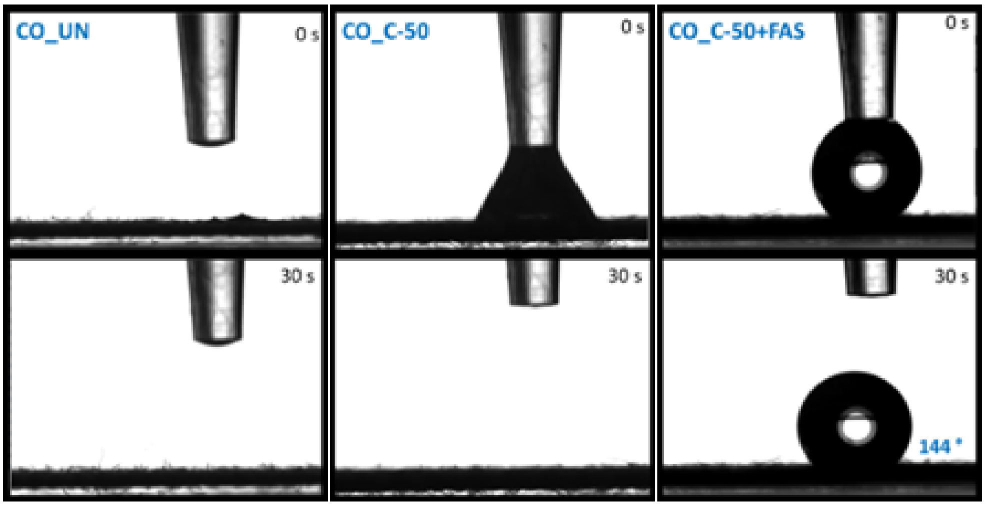

Figure S3. Sessile drop images of the CO_UN, CO_C(50) and CO_C(50)+FAS samples at 0 s and 30 s after contact with the samples.

Supplementary Note 3 |Dynamic water contact angles

We agree that dynamic contact angles are important for passive antibacterial activity, where bacterial adhesion plays an important role. When applied, our coatings exhibit sliding angles of water below 5°. After washing, the textile fibres get damaged by the mechanical force due to rubbing and the pining effect begins. This phenomenon was even worse after several washing cycles, although the static contact angle was still high.

---

[2] Carvalho de Oliveira et al. *ACS Applied Materials & Interfaces* **2023,** *15* (5), 6548-6560. DOI: 10.1021/acsami.2c21011

[3] Zhang et al. *Front. Chem.*, **2021**, 9, 630969. DOI: 10.3389/fchem.2021.630969

[4] Liu et al. Small, **2011**, 7: 2775-2785. DOI: 10.1002/smll.201100651

Supplementary Note 4 | Cytotoxicity

Numerous biological processes are highly dependent on trace metals. Nonetheless, it has been proven that their presence in larger quantities has negative effects on biological systems through interactions or complexes with macromolecular cellular components. Cytotoxicity is often associated with nanoparticles, as it is proven that small particles penetrate deeper into our cells. In our case, we use micro flakes. We agree that if it is harmful to bacteria, it should be also harmful to us if the concentration is high enough. As you can see in Fig. S4, where we evaluate the Co_C(50) coating, there is a small reduction of cells on the right side (extract of the coating) in contrast to the control (just cotton) left side.

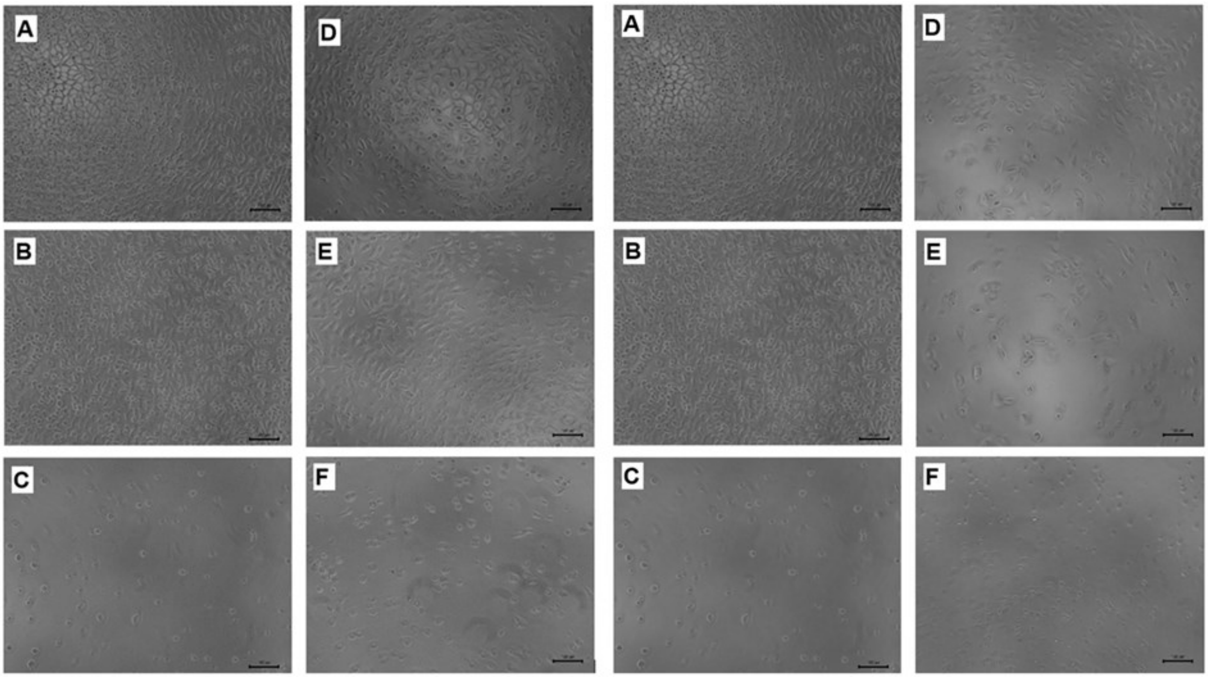

Figure S4. The cells exposed to fresh control media (A - negative control), medium left for 24 ± 1 hours at 37 ± 1°C (B – vehicle control) positive control (C; etoposide 100 µg/mL), and the test item »control cotton (left) and cotton with (Co_C(50) coating (right)« extract (D – 25 %, E - 50 %, F - 100 v/v), after 48 ± 1 hours of exposure (magnification 10 x 20).

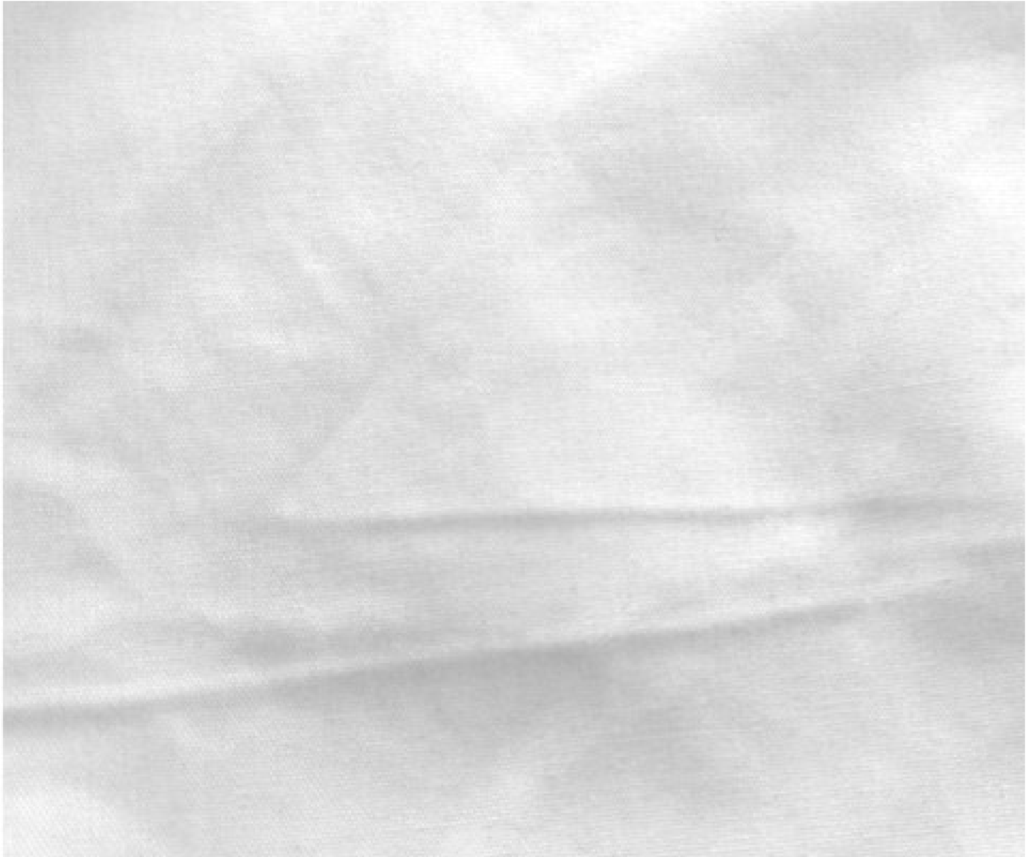

Figure S5. Photograph of cotton textile after anti-pathogen treatment.

Supplementary Note 5 | Washability, an essential feature for satisfying key customers

It is well-accepted that the washing procedure alters the structure of the fibres and the coatings on the textiles.[5] It is similar in the case of antipathogen quality. Naturally, the dissolution decreases with time as a consequence of material (Cu/Ag/FLG) loss reachable for the transfer to the surroundings or/and passivation of material by insolating compounds or/and too slow diffusion to the testing media.

Mathematical modelling is a useful tool for elucidating mechanisms of action to gain insights into antipathogen features. Most investigations reveal that dissolution proceeds according to first-order kinetics; however, this equation does not account for design features of the NP (such as size), making it unable to foretell how alterations to these parameters will affect dissolution. The "shrinking core" model is used there.

Since we only tested a solution containing a mixture of the active components (Cu/Ag/FLG), our dissolving tests have no resemblance to real-world conditions, but you can see from **Fig.**

[5] Volgareet al. *Sci Rep* **2021**, **11**, 19479. DOI: 10.1038/s41598-021-98836-6

S5 below that over time, the concentration of the active species (Cu ions) rises to a peak, then declines as passivation takes place.

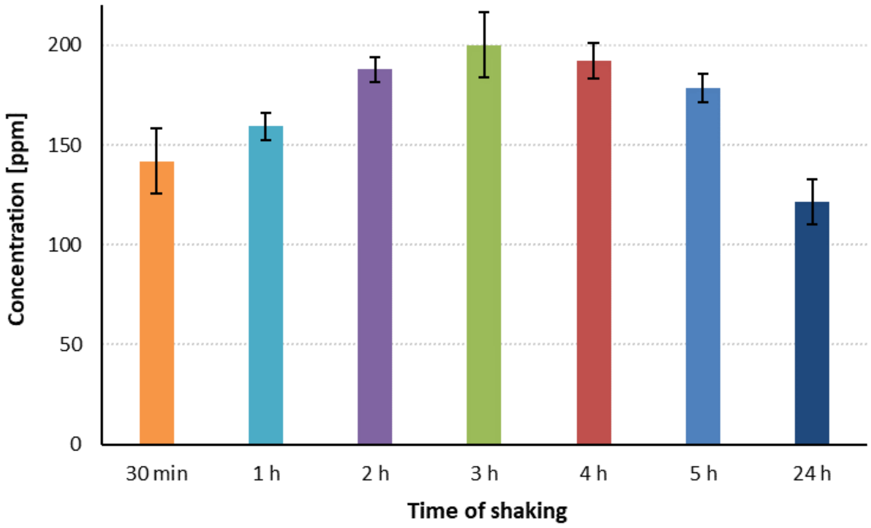

Figure S6. Graphical presentation of Cu ions concentration in solution as a function of time (determined by ICP-MS).

In addition to as prepared samples antimicrobial tests were performed for washed samples. The washing fastness of finished fabrics was determined in an AATCC Atlas Launder-O-Meter Standard Instrument (ISO 105-C01:1989(E) standard method), in which one wash corresponds to five home washings. Washing's effects on coatings from an antipathogen perspective (*E. coli* and *S. aureus*) are seen in **Fig. S6**. When compared to a control sample, the treated sample completely inhibits bacterial growth. After repetitive washings, our coating still inhibits the growth of *E. coli*.

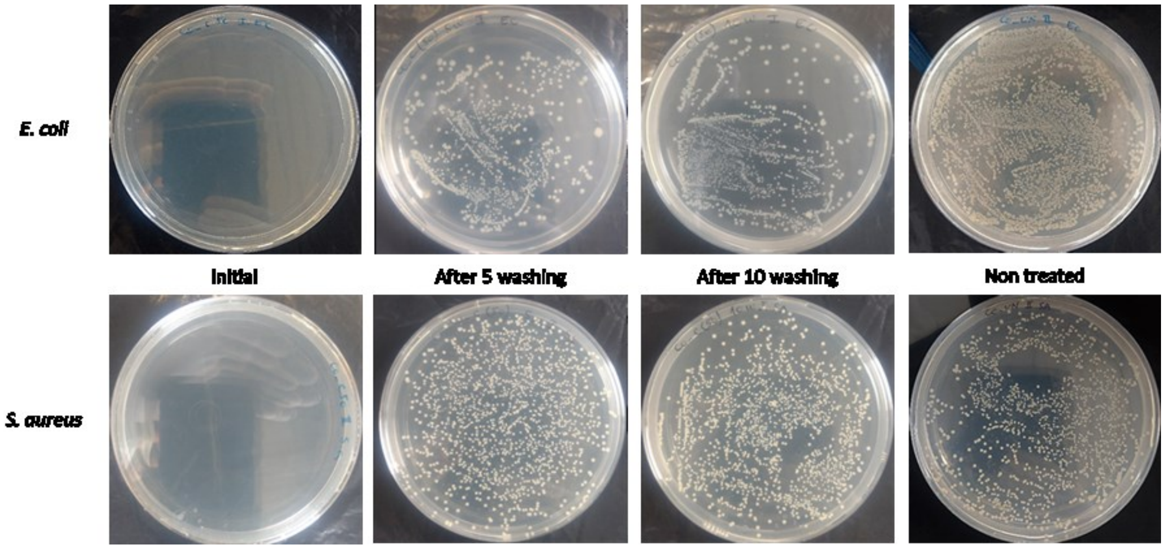

Figure S7. Photos of agar plates, representing the activity of the coatings (Co_C(50)) against E. coli and S. aureus after repetitive washing, shown as bacterial colonies grown on agar plates inoculated with a bacterial suspension shaken with the studied samples for 2 hours and exposed to optimal growth conditions.

Supplementary Note 6 | Credibility of biocidal activity testing

Different antiviral/antibacterial protocols can be used to evaluate the biocidal activity of materials, depending on various factors such as the type of microorganism and the material. An important obstacle currently hindering the development of effective antibacterial textiles is the lack of comparability between different studies, as different tests are used to determine bacterial infectivity, e.g., measurement of optical density at 600 nm, a colorimetric test based on MTT (3-[4, 5-dimethylthiazol-2-yl]-2, 5-bromide), agar diffusion assay, and colony formation. In addition, bacterial infectivity is sometimes determined after cultivation in media containing textile samples and in other cases after recovery from textiles. To determine the most appropriate test system for each type of antibacterial coating, several factors must be considered, such as the choice of appropriate endpoints for passive or active antibacterial activity analysis. While the different approaches produced equivalent results, which approach is most appropriate depends on the details of each analysis.[6] Similar challenges exist when working with viruses, so the most appropriate approaches must be determined here as well.

Antiviral tests were performed at the National Institute of Biology (NIB), while antibacterial tests were performed at NIC in collaboration with the NIB. NIB is is authorized by the Administration of the Republic of Slovenia for Food Safety, Veterinary Sector and Plant Protection as an official laboratory for performing analyzes of plant pathogens, including bacteria and viruses, and is a partner in two European Union Reference Laboratories for bacteriology and for viruses. NIB also operates according to the standard ISO /IEC 17025, which is why we recognize our results as credible.

[6] Haase et al. PLoS ONE 2017, 12 (11): e0188304. DOI: 10.1371/journal.pone.0188304
